# Altered Glycosylation of PSA in Prostate Cancer Tissue

**DOI:** 10.1002/pros.70014

**Published:** 2025-07-09

**Authors:** Hannu Koistinen, Ruusu‐Maaria Merivirta, Timo‐Pekka Lehto, Anna Lempiäinen, Andrew Erickson, Ulf‐Håkan Stenman, Antti Rannikko, Tuomas Mirtti

**Affiliations:** ^1^ Department of Clinical Chemistry and Haematology University of Helsinki and Helsinki University Hospital Helsinki Finland; ^2^ Research Program in Systems Oncology University of Helsinki Helsinki Finland; ^3^ Department of Pathology University of Helsinki and Helsinki University Hospital Helsinki Finland; ^4^ Department of Urology University of Helsinki and Helsinki University Hospital Helsinki Finland; ^5^ iCAN Digital Precision Cancer Medicine Flagship University of Helsinki and Helsinki University Hospital Helsinki Finland; ^6^ Finnish Cancer Institute Helsinki Finland

**Keywords:** glycosylation, in situ proximity ligation, lectin, prostate cancer, PSA

## Abstract

**Background:**

The glycosylation of proteins is commonly altered in cancer. This offers novel opportunities for cancer biomarker development. As prostate‐specific antigen (PSA) is a glycoprotein, identification of cancer‐specific PSA‐glycoforms is feasible. Such PSA‐glycoforms may provide valuable diagnostic and prognostic information.

**Methods:**

PSA‐glycoforms were studied in tissues, using in situ proximity‐ligation assay (PLA)‐based detection with a PSA‐specific antibody and 25 different glycan‐binding lectins.

**Results:**

Using 25 different lectins and a small tissue microarray (TMA), we showed that glycosylation of PSA in cancerous tissues is different from that in benign prostate. In a larger TMA with samples from 162 patients, PSA‐glycoforms detected by succinylated wheat germ (WGA_succ_) and *Vicia villosa* (VVA) lectins were enriched in cancerous tissues as compared to adjacent benign tissues from the same patients (*p* < 10^−4^ for both, Kruskal–Wallis H test), although strong total PSA staining was more often found in benign tissues (*p* = 6*10^−14^).

**Conclusions:**

We showed here that glycosylation of PSA is changed in situ in prostate cancer. The identified lectins, showing preferential binding to cancer‐associated PSA‐glycoforms, will aid the future development of a diagnostic serum test of prostate cancer. Such a test has potential to revolutionize prostate cancer diagnostics.

## 1 Introduction

Prostate‐specific antigen (PSA) blood test is often able to identify prostate cancers at an early stage and PSA‐based screening has been found to reduce prostate cancer‐specific mortality [1, 2, 3]. However, PSA levels in blood circulation can increase in men with benign prostatic hyperplasia (BPH) or prostatitis as well as in patients with clinically insignificant cancers [1, 2, 3, 4, 5]. The diagnostic accuracy of the PSA test can be improved by analyzing total PSA jointly with other biomarkers or different PSA‐isoforms, such as free PSA [6, 7]. Further refinement is needed to improve differentiation between prostate cancer and benign prostatic conditions, and identification of clinically significant prostate cancers.

Glycan structures of glycoproteins are frequently altered in cancer, especially due to cancer‐associated changes in the expression of glycosidases and glycosyltransferases [8, 9, 10, 11, 12]. Such glycans play fundamental roles in tumor development and cancer progression [10, 12, 13]. Since PSA is a glycoprotein, detection of different PSA‐glycoforms in serum or urine has been suggested to provide valuable diagnostic and prognostic information (reviewed in [14, 15, 16, 17, 18, 19, 20, 21]). However, many of these studies have used study cohorts of limited sample sizes which poorly represent the patient groups with the highest clinical need for such a marker, that is those with clinically hard‐to‐predict intermediate‐risk disease. Nevertheless, a recent study [22] reported that glycoprofiling of free PSA in serum samples has potential for early detection of prostate cancers, outperforming total PSA and PSA‐based Prostate Health Index (PHI) tests.

While some studies have confirmed aberrant glycosylation of PSA in cancer tissue by analyzing proteins extracted from tumor tissues [23, 24], most studies have not established whether differentially glycosylated PSA originates from cancerous or benign cells. We recently established a novel in situ proximity ligation assay (PLA)‐based method for detection of specific protein‐glycoforms in tissue sections [25]. With this, we showed that endometrial carcinoma–associated glycoform of glycodelin, which we originally identified by proteomic techniques, is exclusively expressed in cancer, not in normal endometrium, although normal secretory phase endometrium showed strong glycodelin staining [25]. The method is based on binding of a protein‐specific antibody and a glycan‐binding lectin close to each other (in this case in the same glycoprotein molecule). The DNA strands attached to a secondary antibody and to a lectin (via biotin‐avidin interaction) allow sensitive in situ detection by rolling‐circle amplification [25, 26]. Here we modified and optimized the method for the detection of PSA‐glycoforms and showed that glycosylation of PSA in prostate cancer tissue is different from that in cancer adjacent to benign prostate. Our study provides unequivocal evidence that the glycosylation of PSA is frequently altered in prostate cancer tissue. These results provide a solid background for design of blood‐ or urine‐based assays for improved prostate cancer detection.

## 2 Materials and Methods

### 2.1 Antibodies and Lectins

Biotinylated lectin from *Wisteria floribunda* (WFA, L‐1516) was obtained from Sigma. Other lectins were from Vector Laboratories as Biotinylated Lectin Kits I, II and III, or biotinylated MAL I (B‐1315‐2), SNA (B‐1305‐2), and AAL (B‐1395‐1) were obtained separately (Table 1). We used in house monoclonal PSA antibody, clone 5C7, which recognizes both free and complexed PSA and does not show significant cross‐reaction with highly similar KLK2 [27]. To remove Fc region containing glycans from the antibody, we produced Fab fragments using the FabULOUS Fab kit (Genovis).

**Table 1:** Demographics of patients included in analysis.

Variable	Case	Control
Sample type, *n* (%)		
RP	78 (100)	84 (100)
Age at RP (year)		
Mean (SD)	61.1 (6.3)	63.6 (5.9)
RP grade group, *n* (%)		
2	22 (28.2)	41 (48.8)
3	41 (52.6)	27 (32.1)
4	15 (19.2)	16 (19.0)
pT stage, *n* (%)		
pT2	24 (30.8)	33 (39.3)
pT3a	24 (30.8)	30 (35.7)
pT3b	30 (38.5)	21 (25.0)
Presurgery PSA (ng/mL)		
Median (IQR)	9.8 (6.2)	9.0 (7.1)
Follow‐up time, years, median (IQR)	10.0 (5.7)	11.2 (6.0)
Metastasis	78	0
PCa‐specific death	51	0
Death from other reasons	4	7

Abbreviations: IQR, interquartile range; PCa, prostate cancer; PSA, prostate‐specific antigen; pT stage, pathological tumor stage according to American Joint Committee on Cancer (AJCC) TNM 8th edition; RP, radical prostatectomy; SD, standard deviation.

### 2.2 Patients and Ethical Considerations

The patients and tissue microarray (TMA) belong to an earlier described cohort [28, 29]. Briefly, the primary case‐control study cohort consisted of 162 patients with grade group (GG) 2‐4 prostate cancer treated by radical prostatectomy in Helsinki University Hospital in 1992–2015. This consisted of aggressive cases (*n* = 78), who developed metastatic disease during the median 11‐year follow‐up. A subset of patients (*n* = 51) also died due to prostate cancer during the follow‐up. Patients with similar clinical characteristics, but without metastasis or prostate cancer‐specific death during the follow‐up (*n* = 84), served as controls. None of the patients received hormonal treatment before the samples were collected at radical prostatectomy. The patient selection and demographics of the patients included in the analyses are detailed in Figure S1 and Table 1, respectively. The study protocol was approved by Helsinki University Hospital Ethical Committee (HUS/1439/2018) and the National Supervisory Agency for Health and Welfare (Dnro V/38176/2018). As per national legislation, no express consent was required from the studied patients, since the studied patient data were registry data.

### 2.3 Tissue Microarrays (TMA)

For initial analyses, we established a small test TMA, consisting of 3 mm in diameter punches from formalin‐fixed paraffin‐embedded (FFPE) blocks. TMA contained four benign tissues adjacent to cancerous prostatic tissue and 23 prostate cancer foci with Gleason grades between 3 and 5. These patients have not been described above. For construction of the full TMA, consisting of patients described above, clinically relevant cancer foci were annotated from the histological slides by an experienced uropathologist (TM) and one or two 1 mm punches from the index cancer foci were sampled. Benign tissue, adjacent to cancerous prostatic tissue, was also sampled from most of the specimens (*n* = 142).

### 2.4 Immunohistochemical Staining of PSA

TMA sections of 3.5 µm were immunostained using monoclonal PSA antibody (5C7). The slides were incubated over‐night at 37°C and 1 h at 56°C before deparaffinization in xylene and rehydration in a decreasing ethanol series. Antigen retrieval, for 20 min at 98°C, was performed with Dako PT Link Pretreatment module (Agilent Technologies) using Dako Envision TM Flex Target Retrieval solution, high pH (Agilent Technologies). The staining was carried out using an Autostainer 480 (LabVision Corporation, Fremont, CA) and Dako REAL Envision Detection system Peroxidase/DAB+, Rabbit/Mouse (Agilent Technologies). Slides were incubated with the primary antibody (5C7, 0.1 µg/mL) for 30 min at room temperature and with secondary antibody (Dako REAL Envision Detection system) for 20 min at room temperature. In negative control sections, the primary antibody was replaced by Mouse IgG control antibody (Vector Laboratories, Newark, CA). Finally, tissues were counterstained with hematoxylin (Histolab, Espoo, Finland), washed with water and mounted using Aquatex (Merck Millipore, Burlington, MA). The slides were scanned with a Pannoramic 250 Flash III slide scanner (3D Histec, Budapest, Hungary), with 20× magnification (NA 0.8).

Staining intensities of the tissue samples were evaluated by two observers (HK and RM). Only the cancerous areas were evaluated in TMA spots presenting cancer tissue and, likewise, only benign areas were evaluated in those assigned as benign. Apart from this classification, staining evaluation was blinded regarding the clinical data of the patients. Staining intensity in the epithelial cells of prostate adenocarcinomas and benign glands was assessed in an ordinal scale as negative (0), weak/moderate (1) or strong (2). Areas with the strongest staining, comprising a minimum of 5% of the total cancerous or benign epithelial area in the spot, were assessed in scoring. When two spots representing same patients and tissue types were available, only the first ones in sequential order were used for analyses. If the first spot was unable to be scored, the second spot was used.

### 2.5 In Situ (PLA®) for PSA Glycoforms

Unless otherwise indicated, *In situ* PLA® was performed using the Duolink® reagents (DUO) essentially according to manufacturer's instructions (Sigma‐Aldrich) and described earlier [25]. Briefly, after deparaffinization as described for PSA immunohistochemistry above, the antigen retrieval was performed in 10 mM citric acid by warming below boiling point in a microwave oven for 10 min. After washing in phosphate buffered saline, pH 7.4 (PBS), endogenous peroxidase activity was blocked by 30 min treatment with 0.3% H_2_O_2_ (DUO82054) at room temperature. After washing twice for 5 min with PBS containing 0.01% Tween‐20 (PBS‐T), potential nonspecific binding sites were blocked for 30 min at 37°C with Carbo‐Free™ Blocking Solution (SP‐5040‐125, Vector laboratories). Biotinylated lectins (10 µg/ml) were diluted in Carbo‐Free™ Blocking Solution and incubated with sections overnight at 37°C, followed by washing twice for 5 min with PBS‐T. Fab fragment of monoclonal mouse antibody (Mab) against PSA (5C7, 3 µg/ml), conjugated with Duolink *In Situ* probemaker PLUS (DUO92009) and streptavidin (2 µg/ml), conjugated with Duolink *In Situ* probemaker MINUS (DUO92010), were added together in Carbo‐Free™ Blocking Solution to the slides. The reaction with probes, ligation, amplification and detection were performed according to the manufacturer's instructions (DUO92012), except we used, as a chromogen, histofine‐N Simple stain AEC‐solution (Nichirei, 415182 F) and for staining of nuclei hematoxylin (Histolab, Espoo, Finland). The slides were scanned and staining was assessed essentially as for PSA, but on an ordinal scale as negative (0), weak (1), moderate (2) or strong (3). For test TMA half scores were also used. The final scores represent averages of the same two evaluators who evaluated the total PSA staining.

### 2.6 Statistical Analyses

Data were analyzed using R, v.4.3.3 (R Development Core Team, Vienna, Austria). Kruskal–Wallis H test and Mann–Whitney U test were used to compare scores between study groups and between cancer spots and adjacent benign tissue. The association of GG and histological scores was compared using Kruska–Wallis H test. Kaplan–Meier estimator was used to study the prognostic value of PSA and its glycoforms for metastasis‐free and prostate cancer‐specific survival. Here, the samples were divided into two groups based on median score. For comparison of test TMA results Wilcoxon signed‐rank test was used. *p*‐values of < 0.05 were considered significant.

## 3 Results

### 3.1 Lectin Screening

**Figure 1:**
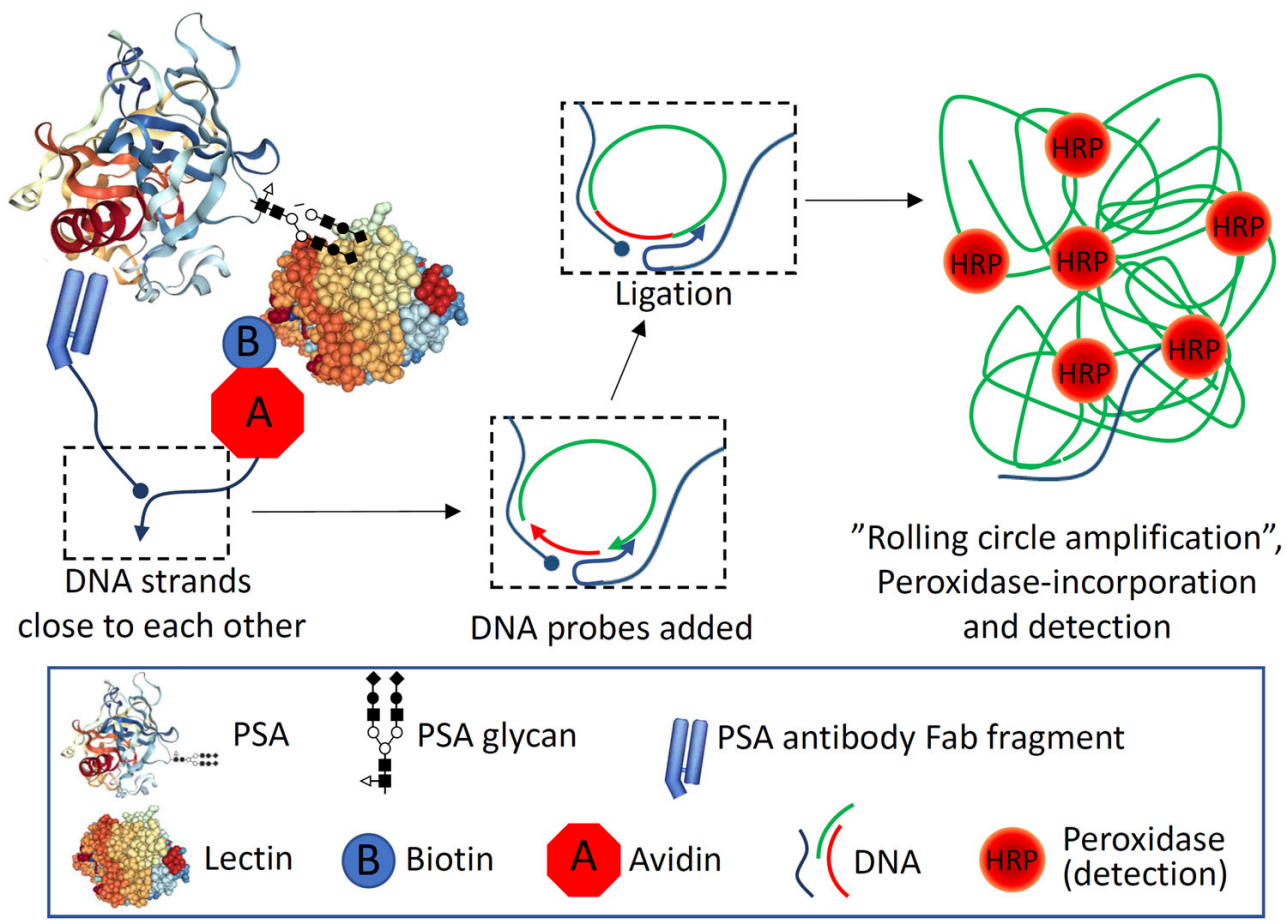
The principle of in situ proximity ligation assay (PLA) of PSA‐glycoforms.

**Figure 2:**
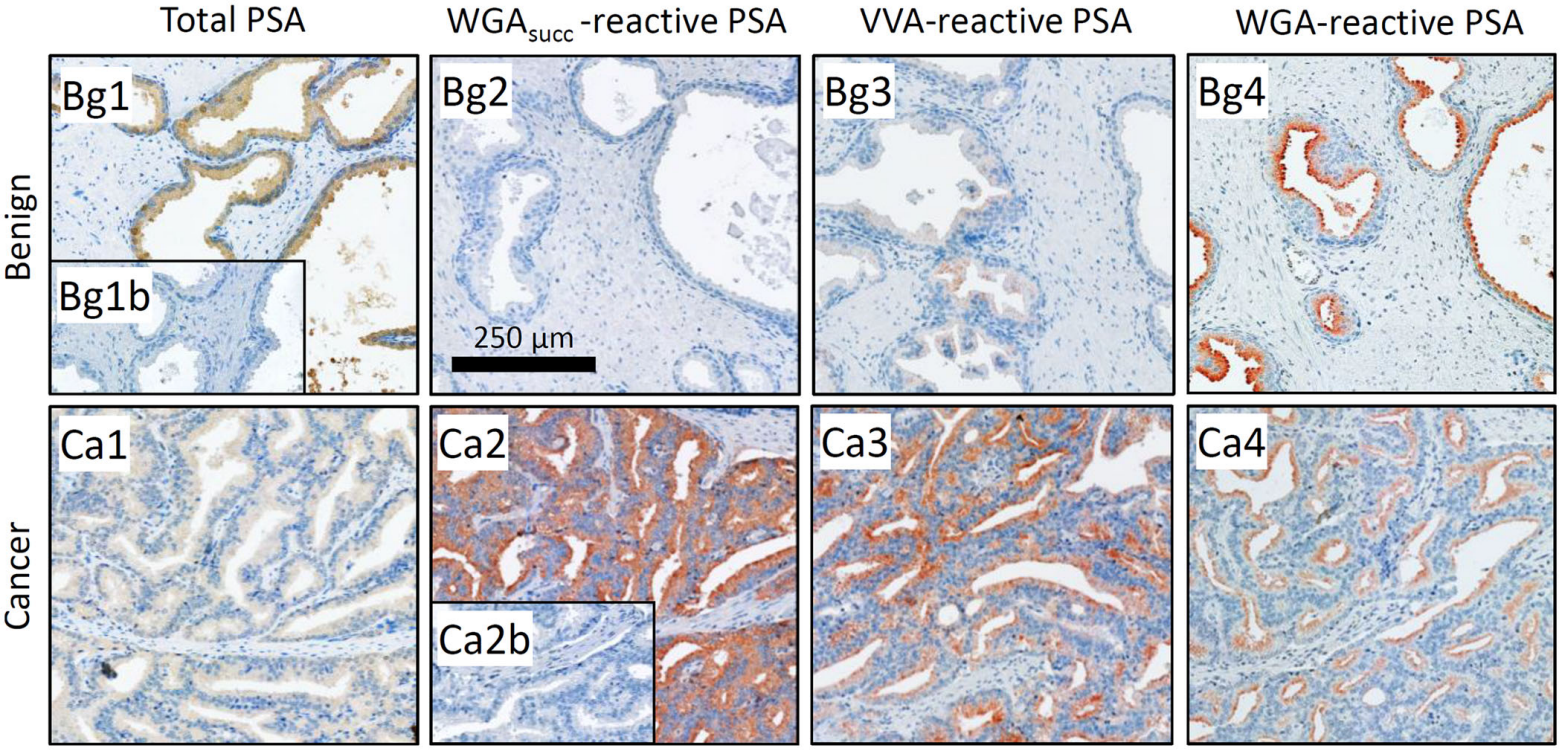
Examples of total PSA (Mab 5C7, figure panels Bg1 and Ca1) and lectin reactive PSA [PLA with Fab fragment of Mab 5C7 and lectins WGA_succ_ (Bg2 and Ca2), VVA (Bg3 and Ca3) and WGA (Bg4 and Ca4)] in prostate cancer (Ca) and benign (Bg) prostatic tissues (same tissues in all). Inset (Bg1b) in total PSA staining of benign tissue is a negative control with nonimmune IgG. Inset (Ca2b) in PLA staining of cancer tissue is PLA staining in which SJA lectin was used instead of WGA_succ_. Scale bar (in Bg2) is 250 µm.

We set up a staining procedure for detection of PSA‐glycoforms in tissue sections (Figure 1). With this method, a small test TMA, consisting of four benign tissues adjacent to cancerous prostatic tissue and 23 prostate cancer foci, was stained using 25 different lectins and a Fab fragment of a PSA‐binding antibody 5C7. Several lectins, in combination with the antibody, showed strong PSA‐specific staining (Table 2, Figure 2), while controls without lectin or without conjugated antibody fragment (the latter with WGA_succ_ lectin) did not show any staining. Especially *Erythrina cristagalli* (ECL), succinylated wheat germ (WGA_succ_), *Griffonia simplicifolia* I (GSL I) and *Vicia villosa* (VVA) lectins showed no or weak staining of PSA in benign samples, while several cancer samples showed strong staining (*p* < 0.05 for all). The staining of total immunoreactive PSA (i.e., 5C7 antibody staining without lectins) was more pronounced in benign tissues than in cancer (*p* = 0.01).

**Table 2:** Histochemical staining of lectin–reactive PSA (in situ proximity ligation, all lectins with Fab fragment of PSA antibody 5C7) and total immunoreactive PSA (5C7) in benign tissue adjacent to cancerous prostatic tissue and prostate adenocarcinoma tissues. Staining in tissues was scored between 0 and 3, with 0.5 increments (average of the scoring by two persons).

Lectin	Abbreviation	Staining [median (IQR)]		*p*‐value^a^	Prefererred specificity^b^
		Benign (*n* = 4)	Cancer (*n* = 23)		
*Sambucus nigra*	SNA	2.8 (0.13)	2.5 (0.75)	0.44	Neu5Acα6Gal/GalNAc
*Erythrina cristagalli*	ECL	0 (0)	1.5 (1.8)	**0.009**	Galβ4GlcNAc
*Maackia Amurensis I*	MAL I	1.8 (0.31)	1.3 (0.9)	0.18	Galβ4GlcNAc
*Ulex europaeus* I	UEA I	1.3 (0.9)	2 (2)	0.21	αFuc
*Aleuria aurantia*	AAL	1.0 (0.5)	1.0 (2.5)	0.89	Fucα6GlcNAc
Concanavalin A	Con‐A	1 (0.5)	1.3 (1.3)	0.54	αMan, αGlc
*Pisum sativum*	PSA	2 (0.44)	1.8 (1.5)	0.89	αMan, αGlc
*Lens culinaris*	LCA	0 (0)	0 (0.75)	0.10	αMan, αGlc
*Ricinus communis* I	RCA I	2.4 (0.94)	2 (1.3)	0.33	Gal
*Griffonia simplicifolia* II	GSL II	1.9 (0.94)	0.5 (2.5)	0.50	αGal
*Griffonia simplicifolia* I	GSL I	0.38 (0.31)	1.3 (1.5)	**0.03**	αGal, αGalNAc
*Dolichos biflorus*	DBA	0.25 (0.88)	1.3 (1.3)	0.27	αGalNAc
Soybean	SBA	0 (0)	0 (1)	0.10	α>βGalNAc
*Sophora japonica*	SJA	0 (0)	0 (0.25)	0.28	βGalNAc
*Vicia villosa*	**VVA**	0.5 (0.25)^c^	2 (1.3)^c^	**0.01**	GalNAc
*Wistera floribunda*	WFA	0.5 (1.3)	2 (2.1)	0.15	GalNAc
Wheat germ	WGA	2.6 (0.38)	1.8 (1.3)	0.06	GlcNAc
Succinylated wheat germ	**WGA**_**succ**_	0 (0.13)	1.5 (1.6)	**0.01**	GlcNAc
*Datura stramonium*	DSL	3 (0.63)	3 (0.88)	0.74	(GlcNAc)_2‐4_
*Lycopersicon esculentum*	LEL	3 (0.63)^c^	2.5 (1)^c^	0.35	(GlcNAc)_2‐4_
*Solanum tuberosum*	STL	3 (0)^c^	3 (0.5)^c^	0.23	(GlcNAc)_2‐4_
Peanut	PNA	1.8 (1)	1.5 (0.63)	0.51	Galβ3GalNAc
Jacalin	Jacalin	1.8 (1.3)^c^	2.5 (0.75)^c^	0.66	Galβ3GalNAc
*Phaseolus vulgaris* Erythroagglutinin	PHA‐E	1.3 (1.7)	2.5 (1.1)	0.07	Galβ4GlcNAcβ2Manα6 (GlcNAcβ4) (GlcNAcβ4Manα3) Manβ4
*Phaseolus vulgaris* Leucoagglutinin	PHA‐L	1 (2)	1.5 (2)	0.70	Galβ4GlcNAcβ6 (GlcNAcβ2Manα3) Manα3
**Antibody**					
Anti PSA, 5C7, 0.1 µg	PSA/5C7	2.9 (0.38)	1.8 (1)	**0.01**	Immunoreactive PSA

*Note: p*‐values < 0.05 are bolded.

^a^ Wilcoxon signed‐rank test.

^b^ Specificity is based on the manufacturer's (Vector Laboratories) information. It is noteworthy that lectins generally have broad specificity, thus, for many of the lectins also several other, often more complex, glycan determinants have been reported and binding affinity towards these may vary significantly [30, 31].

^c^ For benign samples *n* = 3 and for Cancer *n* = 21.

Based on the preliminary experiments above we selected three of the lectins showing the most pronounced difference in PSA‐glycoform staining between cancer and adjacent benign tissues, that is, ECL, WGA_succ_, and VVA, for PSA‐glycoform stainings in a larger prostate cancer TMA, consisting of cancer and adjacent benign tissue samples from patients with Grade Group 2–4 prostate cancers. Here strong staining of ECL reactive PSA was found, in addition to cancerous tissues, in some of the benign tissues adjacent to cancer and was not further evaluated.

**Figure 3:**
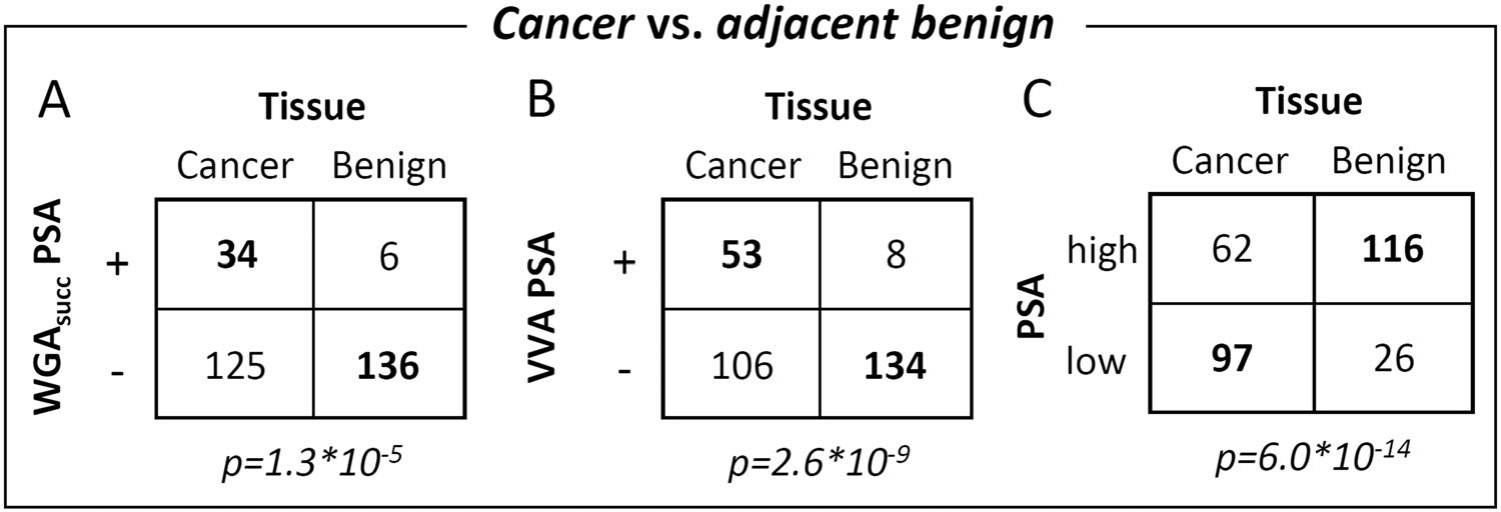
Comparison of (A) WGA_succ_ and (B) VVA reactive PSA (in situ PLA with Fab fragment of PSA‐antibody and lectins) and (C) total PSA staining (with antibody) in cancerous and adjacent benign prostate. The number of cancer and adjacent benign samples with positive (+) and negative (−) staining (or in case of total PSA high or low staining) are shown. The corresponding Kruskal–Wallis H test *p*‐values are shown. −, staining score 0; +, staining score 1–3; high, staining score 2; low, staining score 0–1.

Contrary to ECL, strong staining (score 3) of WGA_succ_ and VVA reactive PSA was undetectable in adjacent benign tissues, while in 5.0% and 6.9% of cancerous tissues strong staining was observed for WGA_succ_ and VVA reactive PSA, respectively. Weaker staining was observed in some benign samples, but the overall staining of WGA_succ_ and VVA reactive PSA was strongly associated with cancer as compared to adjacent benign tissues (*p* < 10^−4^ and *p* < 10^−8^, respectively) (Figure 3). In contrast to these PSA‐glycoforms, total PSA staining (antibody 5C7) was observed in almost all samples (95.6%) and was stronger in adjacent benign tissues than in cancer (*p* < 10^−13^) (Figure 3).

While the studied PSA‐glycoforms were clearly associated with cancer, there was no significant association with Grade Group or metastasis‐free or prostate cancer‐specific survival (*p* > 0.05 for all). Total PSA staining was stronger in samples from patients with lower Grade Group cancers (*p* = 0.02). Furthermore, weak PSA staining in cancer‐adjacent benign tissue tended to be associated with poor metastasis‐free survival (*p* = 0.055), while in cancer tissue no such association was found (*p* = 0.26).

## 4 Discussion

While PSA tests, when used properly, provide highly useful clinical information, widespread use of opportunistic PSA tests or nonrestricted screening lead to a significant number of unnecessary biopsies and overdiagnosis [32, 33]. One potential approach to improve the diagnostic accuracy of PSA test is to assess different PSA‐glycoforms. To aid the development of better noninvasive tests for prostate cancer detection, we show here, for the first time in situ, that glycosylation of PSA is changed in cancer tissue as compared to adjacent benign prostate.

For the detection of PSA‐glycoforms, we used a similar in situ proximity‐ligation ‐based method we have previously established for another glycoprotein, glycodelin [25]. This method allows glycoform specific detection of a given glycoprotein, based on binding of a protein‐specific antibody and a glycan‐binding lectin close to each other, for example, in the same glycoprotein. Previously several lectins have been suggested to selectively detect prostate cancer associated PSA, especially in serum samples (reviewed in [14, 15, 16, 17, 18, 19, 20, 21]). Many of these studies have utilized lectins from *Wisteria floribunda* (WFA) [22, 34, 35, 36] and *Maackia amurensis* (MAL) [37, 38, 39], recognizing carbohydrate epitopes like LacdiNAc/terminal GalNAc and α2,3‐sialic acid, respectively [30, 40, 41]. The studies with MAL lectin have been complemented with those using antibody recognizing α2,3‐linked sialic acid [42]. While several of these studies showed that determination of these PSA‐glycoforms improves the accuracy of prostate cancer detection as compared to conventional PSA tests (total and free PSA and PSA density) [22, 34, 35, 36, 37], the improvements have been relatively modest and not found in all studies [43]. Our approach should allow selection of lectins that are able to better distinguish between cancer and benign conditions.

Here we found in preliminary studies using a small test TMA, that while there was a trend towards increased staining of WFA‐reactive PSA in cancerous prostate, some of the benign samples also showed strong staining of this PSA‐glycoform. Furthermore, staining of MAL I‐reactive PSA was similar both in cancerous and benign prostate. Thus, based on our results, WFA‐ and MAL I ‐reactive PSA‐glycoforms are not optimal markers for prostate cancer detection, although direct comparison of tissue and serum glycoforms may be hampered, *e.g*., by the presence of glycosidases in blood circulation [44]. Instead, our results suggest that PSA‐glycoforms that react with succinylated wheat germ lectin (WGA_succ_) and *Vicia villosa* lectin (VVA) are highly enriched in cancerous prostate as compared to adjacent benign prostatic tissue, irrespective of the fact that in benign prostate the total PSA staining was more pronounced. This was confirmed with a larger patient cohort with samples from patients with GG2‐4 cancers, in which current biomarkers or even histological evaluation of diagnostic biopsies are not able to sufficiently risk stratify patients [45]. To our knowledge, these lectins have not been previously used for detection of PSA‐glycoforms. WGA_succ_ and VVA recognize epitopes with GlcNAc and terminal GalNAc, respectively [30, 46, 47].

While according to monosaccharide binding preference it would have been expected that PSA‐glycoform stainings with VVA and WFA or WGA and WGA_succ_ would be similar, those showed marked differences. This is in keeping with the observation that several lectins, showing apparently similar monosaccharide specificities, have different affinities for complex glycans [30, 31]. However, the comparison of stainings was performed using a very small number of samples and we, indeed, found a positive relationship between staining intensities of VVA‐ and WFA‐reactive PSA (results not shown).

Often the glycosylation changes in cancer have been ascribed to altered expression of enzymes involved in glycosylation [9, 12]. Unlike PSA, most of these enzymes are intracellular and therefore not detectable by serological tests. For the detection of cancer, it is more practical to study cancer‐associated glycoforms of proteins secreted from cancer cells and present in blood circulation or urine, rather than intracellular enzymes responsible for altered glycosylation. It is noteworthy that different proteins in a tissue can carry similar glycans, but may also be very differently glycosylated or even the different glycosylation sites of a given protein may carry very different glycans [48]. Furthermore, individual heterogeneity in glycosylation of a given protein can be observed within a single tissue and interindividual variation is common, as exemplified by ABO(H) blood groups [49, 50]. In case of PSA, also prostate cancer‐associated genetic polymorphism (SNP) (with an allele frequency of 4%) that leads to an additional glycosylation site in PSA has been reported [51]. These sources of variation complicate the use of glycan‐based biomarkers and perhaps, at least partly, explain why not all PSA‐positive cancer tissues were not positive for stained PSA‐glycoforms. To note, we did not find any association between WGA_succ_ or VVA lectin reactive PSA and patients ABO blood group (results not shown).

The antibody (5C7) we used for the stainings recognize both free and complexed PSA [27]. Since a small fraction of PSA is complexed with α_1_‐antichymotrypsin (ACT, serpinA3) already in prostate [52] we can't rule out the possibility that the lectin binds to glycans of ACT in complex with PSA rather than glycans of PSA. In blood circulation most of the PSA is complexed with ACT [53]. Therefore, we anticipate that the translation of the findings to a useful serological test is easier for total PSA (free and complexed PSA). It should be noted that in prostate the PSA‐ACT complex is likely to contain ACT expressed in prostate, while in blood circulation the majority of ACT is likely derived from the liver and, thus, may be differently glycosylated [54].

The approach we have used here could be applied also for other cancers and markers than prostate cancer and PSA. Here it is noteworthy that not all antibodies that function well in conventional immunoassays are suitable for in situ proximity ligation, where the two probes need to be close enough to each other to allow ligation of DNA strands. Furthermore, we have found that several lectins bind directly to antibodies, which are glycoproteins, and this varies even between different in‐house antibodies, produced using identical methods. Such binding causes high background signal in assays. To get rid of the major glycans of the antibody, i.e., those in Fc region, we used here antibody Fab fragments. Such fragments may also contain glycans [55]. However, this is not likely to explain our results with WGA_succ_ and VVA lectins since, in in situ proximity ligation, those showed much more pronounced staining in cancer as compared to adjacent benign tissue, while opposite was observed for total PSA staining.

## 5 Conclusions

Our results further prove that glycosylation of PSA is changed in prostate cancer and identify lectins that can be used in conjunction with a PSA‐antibody for the establishment of a serological diagnostic assay selective for cancer‐associated PSA‐glycoforms. We foresee that such an assay would significantly reduce the number of diagnostic biopsies carried out for patients with benign conditions, while, based on our results, it may not provide prognostic information. To our knowledge, this is the first time PSA‐glycoforms have been studied in situ, especially using a reasonable sized patient cohort, which, furthermore, consists of patients with cancers showing mid spectrum of differentiation (GG2 to 4).

## Author Contributions

Hannu Koistinen and Tuomas Mirtti planned and designed the project. Hannu Koistinen drafted the manuscript with help from other co‐authors. Hannu Koistinen, Ruusu‐Maaria Merivirta and Tuomas Mirtti reviewed and scored the pathological samples. Timo‐Pekka Lehto performed statistical analyses. Timo‐Pekka Lehto, Andrew Erickson, Antti Rannikko and Tuomas Mirtti collected patient samples and clinical information, and participated in TMA construction. All authors played a role in interpreting the results, and read, commented, and approved the final version of the manuscript.

## Ethics Statement

The study protocol was approved by Helsinki University Hospital Ethical Committee (HUS/1439/2018) and the National Supervisory Agency for Health and Welfare (Dnro V/38176/2018). As per national legislation, no express consent was required from the studied patients, since the studied patient data were registry data.

## Conflicts of Interest

The authors declare no conflicts of interest.

## Supporting information

Supplementary Figure1.

## Data Availability

Biomarker data are available on request from the corresponding author.
