# Supplementary material for: Altered Glycosylation of PSA in Prostate Cancer Tissue
Source: Prostate. 2025 Jul 9;85(14):1290–8. doi: 10.1002/pros.70014 (PMC12379856; doi:10.1002/pros.70014)
Supplement: Supplementary file 1 — Supplementary Figure1. [file PROS-85-1290-s001.pdf]

Supplementary Figure 1

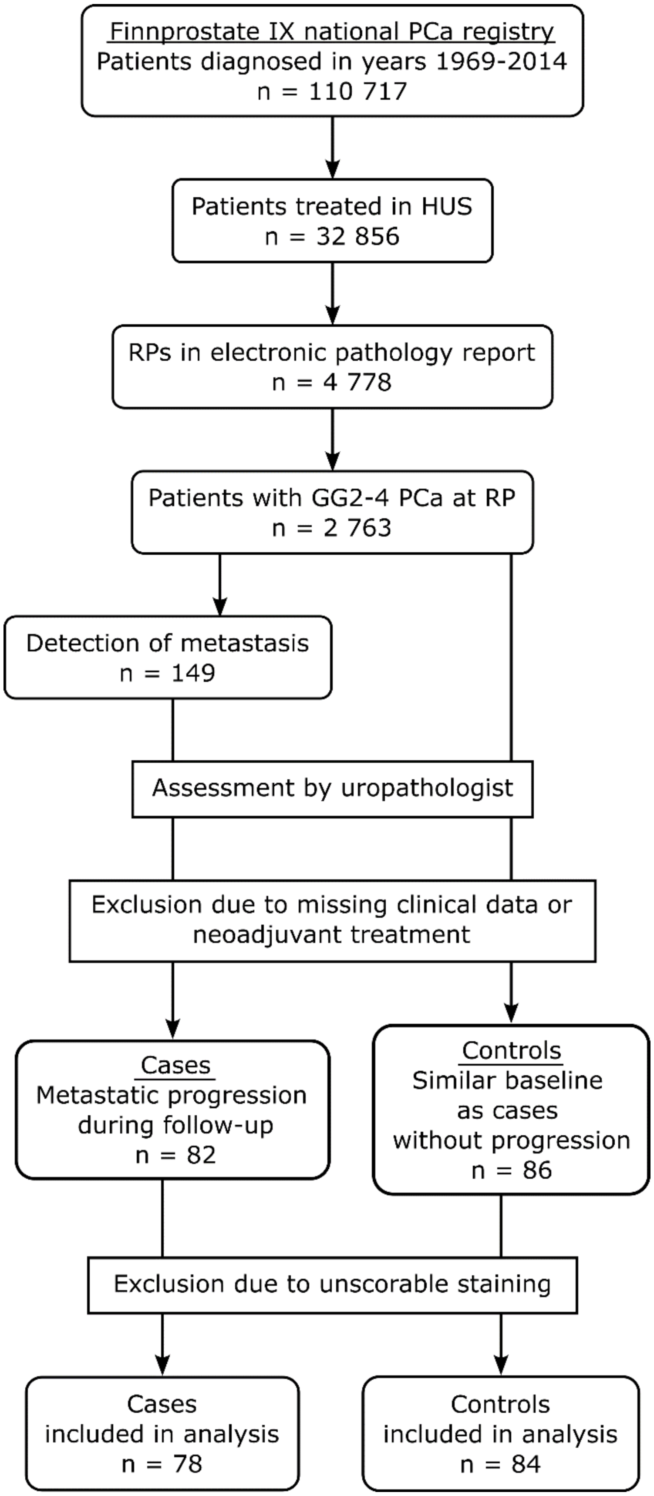

Supplementary Figure 1. Patient selection. Abbreviations: GG, Grade Group; HUS, Helsinki University Hospital; PCa, prostate cancer; RP, radical prostatectomy.
